# Relationship between Lifetime Suicide Attempts and Schizotypal Traits in Patients with Schizophrenia

**DOI:** 10.1371/journal.pone.0107739

**Published:** 2014-09-16

**Authors:** Toshiya Teraishi, Hiroaki Hori, Daimei Sasayama, Junko Matsuo, Shintaro Ogawa, Ikki Ishida, Anna Nagashima, Yukiko Kinoshita, Miho Ota, Kotaro Hattori, Hiroshi Kunugi

**Affiliations:** Department of Mental Disorder Research, National Institute of Neuroscience, National Center of Neurology and Psychiatry, Tokyo, Japan; Chiba University Center for Forensic Mental Health, Japan

## Abstract

Patients with schizophrenia are at increased risk for suicide. Various risk factors for suicide have been reported in schizophrenia; however, few studies have examined the association between personality traits and suicidal behavior. We administered the Schizotypal Personality Questionnaire (SPQ) to 87 Japanese patients with schizophrenia (49 males; mean age 38.1±10.6 years) with and without a history of suicide attempts (SA and nSA groups, respectively), and 322 controls (158 males; mean age 40.8±13.9 years). As expected, an analysis of covariance (ANCOVA) controlling for age and sex showed that all SPQ indices (total SPQ score and all three factors, i.e., cognitive-perceptual, interpersonal, and disorganized) were significantly higher in patients with schizophrenia (SA+nSA groups), than controls (p<0.001 for all comparisons). Furthermore, there were significant differences in the total score and the interpersonal and disorganized factors between the SA and nSA groups (nSA<SA, p<0.01 for all comparisons). Receiver operating characteristic analysis showed that a total SPQ score of 33.5 was the optimal cut-off value to discriminate the SA group from the nSA group (χ^2^[1] = 10.6, p = 0.002, odds ratio: 4.7, 95% confidence interval: 1.8–12.1, sensitivity: 0.70, specificity: 0.67). These results suggest that high schizotypy is associated with lifetime suicide attempts, and that the total SPQ score might be useful to assess the risk of suicide attempt in patients with schizophrenia.

## Introduction

Suicide in patients with schizophrenia has long been a major topic of research because the suicide mortality rate is higher in this group than in the general population. The lifetime suicide rate among patients with schizophrenia is estimated to be 4% to 10% [1]–[3]. Consistently reported suicide risk factors among schizophrenic individuals are male gender, poor adherence to treatment, greater insight into the illness, depressive episodes, substance abuse/dependence, and previous suicide attempts [4]–[6]. Protective factors such as defect state at an early stage and having daily activities have been reported [5]. In a meta-analysis [4], hallucination was found to be a protective factor, although conflicting negative results have also been reported [6].

Suicide attempt is one of the most frequently reported risk factors associated with suicide in patients with schizophrenia [4], with odds ratios of 10.3 [7] and 5.0 [8]. The risk factors for suicide attempts in patients with schizophrenia are depressive episodes, hopelessness, insight into the illness [9], and alcohol abuse/dependence [10]. In a twin study, common and unique environmental factors accounted for 60% and 40% of the variance in suicide attempts in schizophrenia, respectively, while the additive genetic factor negligibly affected suicide attempts [11]. A large epidemiological study revealed that higher education, female gender and single marital status were associated with suicide attempts in patients with first-episode schizophrenia [12].

Personality has been the focus of research on suicidal behavior in various populations: antisocial personality disorder, which is correlated with Factor 2 of the Psychopathy Checklist–Revised, has been studied in a prison population [13]; neuroticism, hopelessness, and an external locus of control, measured by the Eysenck Personality Questionnaire (EPQ), Beck Hopelessness Scale, and Locus of Control of Behavior scale, respectively, have been examined in a young population [14]; and aggressiveness assessed by the Freiburg Personality Inventory was studied in a military population [15]. Harm avoidance (HA), as measured by the Temperament and Character Inventory (TCI) has repeatedly been associated with suicidal behavior in non-clinical and clinical populations [16]–[18]. With respect to patients with schizophrenia, HA and persistence (PS) were positively associated with suicidal behavior, while self-directedness (SD) and cooperativeness (CO) were negatively associated with suicidal behavior [19]. However, studies examining such relationships in schizophrenia are still scarce.

The DSM categorical diagnosis of schizotypal personality disorder (SPD) was also found to increase the risk for suicide attempts [20]. Based on the DSM-III criteria for SPD, the Schizotypal Personality Questionnaire (SPQ) was developed by Raine [21] to assess schizotypal personality traits or proneness to psychosis in the general population. The three-factor model measured by the SPQ, consisting of cognitive-perceptual, interpersonal, and disorganized factors, was shown to fit the data in patients with schizophrenia, as well as in the nonclinical population [22]. This supports the dimensional model of schizotypy, ranging from the general population to patients with schizophrenia. Accordingly, our group reported that high schizotypy assessed with the SPQ was associated with poorer neurocognitive functions in a non-clinical population [23], [24]. It is therefore feasible to examine the associations between the SPQ and symptoms, characteristics, and behaviors in schizophrenia. However, few studies have examined these associations [22], [25], [26], and, to our knowledge, no study has examined the relationship between the SPQ and suicidality in schizophrenia.

The main purpose of this study was to examine the association between schizotypy assessed with the SPQ and suicide attempts. We hypothesized that high schizotypy would be associated with suicide attempts.

## Methods

### Participants

The participants were 87 patients with schizophrenia and 322 healthy volunteers, matched for age and sex. All participants were non-elderly individuals (≤65 years old) and biologically unrelated Japanese who resided in the western part of metropolitan Tokyo. They were recruited through advertisements in free local magazines, by word of mouth, and from our website announcement. Patients were also recruited from the National Center of Neurology and Psychiatry (NCNP) Hospital, Tokyo, Japan. Consensus diagnoses were made by at least two experienced psychiatrists according to the Diagnostic and Statistical Manual of Mental Disorders, 4^th^ edition (DSM-IV) criteria [27] for schizophrenia, based on the Japanese version of the Mini-International Neuropsychiatric Interview (M.I.N.I.) [28], [29] and all available information from observations, additional unstructured interviews, and medical records. Healthy volunteers were interviewed by psychiatrists using the M.I.N.I. and additional unstructured interviews to confirm that they had no history of psychiatric illness, contact with psychiatric services, or suicidal behavior. Participants were excluded from both the patient and control group if they met the DSM-IV criteria for mental retardation or substance dependence or abuse, had a history of severe head injury, or had a serious physical disorder. The present study was conducted in accordance with the Declaration of Helsinki. The capacity of patients to understand the nature and purpose of this study and to consent was confirmed by their psychiatrists, based on their medical charts, or using unstructured interviews conducted by two experienced psychiatrists. Patients' legal next of kin consented on behalf of patients who did not have the capacity. After explaining the nature of the study procedures, written informed consent was obtained from all participants or their next of kin. The study was approved by the ethics committee of the NCNP, Japan.

### Clinical evaluation and antipsychotic medication

Clinical symptoms were assessed by experienced psychiatrists using the Japanese version of the Positive and Negative Syndrome Scale (PANSS) [30], [31]. Patients were divided into two groups: those with and without a history of suicide attempts (SA and nSA, respectively). Suicide attempt was defined as non-fatal, potentially self-injurious behavior with the intent to die, which may or may not result in injury [32]. Information on family history of any psychiatric disorder and psychiatric service use among patients' first- and second-degree relatives was obtained from all patients. These clinical evaluations were performed by clinical psychologists or psychiatrists using unstructured interviews and the patients' medical records, if available. All patients were clinically stable at the time of the interview and were able to complete the self-report questionnaire described below. Their daily dose of antipsychotics was calculated as chlorpromazine equivalents (CPZeq) in mg/day according to published guidelines [33], [34].

### Schizotypal personality assessment

The Schizotypal Personality Questionnaire (SPQ) was distributed to all participants. Each participant was allowed to take as much time as needed to complete the questionnaire. The SPQ [21] is a 74-item dichotomous (yes/no) self-report questionnaire that incorporates all nine symptoms of the DSM-III-R [35] criteria for the diagnosis of schizotypal personality disorder (SPD). All items with an answer of “yes” are scored 1. There are nine subscales, which have been found to load onto three factors: cognitive-perceptual (“ideas of reference,” “odd beliefs/magical thinking,” “unusual perceptual experiences,” and “suspiciousness/paranoid ideation” subscales), interpersonal (“social anxiety,” “no close friends,” “constricted affect,” and “suspiciousness/paranoid ideation” subscales), and disorganized (“eccentric/odd behavior and appearance” and “odd speech” subscales) [36]. The Japanese version of the SPQ, validated by Fujiwara et al. [37], was used in this study.

### Statistical analysis

Averages are reported as mean ± standard deviation (S.D.). For categorical variables, between-group comparisons were conducted using χ^2^ tests. Group differences in continuous demographic variables were examined using *t*-tests or an analysis of variance (ANOVA). Cronbach's α was calculated to assess the internal consistency of the total score of the SPQ and each of its three factors. An analysis of covariance (ANCOVA) controlling for age and sex was used to compare SPQ indices between the two groups. Cohen's *d*, Cramer's *V*, *η* ^2^, and partial *η* ^2^ were calculated as measures of effect size for *t*-tests, χ^2^ tests, ANOVAs, and ANCOVAs, respectively. Receiver operating characteristic (ROC) analysis was performed to determine the total SPQ cut-off value that best discriminated between the SA and nSA groups. Statistical significance was set at p<0.05 (two-tailed). Statistical Package for the Social Sciences (SPSS) version 11.0 (SPSS Japan, Tokyo) was used for the analyses.

## Results

### Demographic and clinical characteristics

The demographic and clinical characteristics of patients and controls are presented in Table 1. Age and sex were not significantly different among the three groups (i.e., SA, nSA, and control groups). Controls had significantly more years of education than patients did. There was no significant difference between the SA and nSA groups for years of education, age of onset, family history of any psychiatric disorder, or antipsychotic medication. The SA and nSA groups did not significantly differ on the total PANSS score or its subscales (i.e., positive, negative, or general subscales).

**Table 1:** Demographic and clinical characteristics of patients with schizophrenia with or without suicide attempts, and healthy controls.

	Patients with schizophrenia			Healthy controls	*t*-test or χ^2^ test		Analysis of variance
	Suicide attempt	No suicide attempt	Total		SA vs. nSA	Total patients vs. controls	SA vs. nSA vs. controls
	(SA; n = 30)	(nSA; n = 57)	(n = 87)	(HC; n = 322)			
Demographics							
Age (year)	39.1±11.3	37.7±10.3	38.1±10.6	40.8±13.9	t(85) = 0.58, p = 0.56, Cohen's *d* = 0.13	t(407) = 1.9, p = 0.054, Cohen's *d* = 0.20	F(2, 406) = 1.5, p = 0.23, *η* ^2^ = 0.01
Sex, male: n (%)	14 (46.7)	35 (61.4)	49 (56.3)	158 (49.1)	?^2^(1) = 1.7, p = 0.26, Cramer's *V* = 0.14	?^2^(1) = 1.4, p = 0.28, Cramer's *V* = 0.06	?^2^(2) = 3.1, p = 0.21, Cramer's *V* = 0.09
Education (year)	13.2±1.6	13.6±2.3	13.5±2.1	15.4±2.6	t(85) = 0.69, p = 0.41, Cohen's *d* = 0.19	t(407) = 6.4, p≤ 0.001, Cohen's *d* = 0.76	F(2, 406) = 20.4, p≤ 0.001, *η* ^2^ = 0.09
Age of onset (year)	22.6±7.9	23.7±7.4	23.3±7.5		t(85) = 0.45, p = 0.51, Cohen's *d* = 0.15		
Family history of any psychiatric disorder, n (%)	18 (60)	22 (38.6)	40 (46.0)		?^2^(1) = 3.6, p = 0.057, Cramer's *V* = 0.20		
Antipsychotic medication							
CPZeq (mg/day)	524.5±392.7	545.5±457.3	538.3±433.9		t(85) = 0.21, p = 0.83, Cohen's *d* = 0.05		
typCPZeq (mg/day)	157.7±249.1	265.9±342.5	228.6±316.2		t(85) = 1.5, p = 0.13, Cohen's *d* = 0.34		
atypCPZeq (mg/day)	366.9±368.3	279.6±425.9	309.7±407.0		t(85) = 0.95, p = 0.35, Cohen's *d* = 0.21		
Receiving only typical antipsychotics: n	8	22	30				
Receiving only atypical antipsychotics: n	14	22	36		?^2^(1) = 1.1, p = 0.43, Cramer's *V* = 0.13		
Symptoms							
The Positive and Negative Syndrome Scale (PANSS)							
Total score	59.9±15.9	55.2±14.8	56.8±15.3		t(85) = 1.4, p = 0.18, Cohen's *d* = 0.31		
Positive subscale	14.2±5.5	13.2±4.8	13.5±5.0		t(85) = 0.87, p = 0.39, Cohen's *d* = 0.20		
Negative subscale	16.2±5.5	14.9±6.3	15.4±6.0		t(85) = 0.95, p = 0.35, Cohen's *d* = 0.22		
General subscale	29.5±8.8	27.1±7.0	27.9±7.7		t(85) = 1.4, p = 0.17, Cohen's *d* = 0.31		

CPZeq: Chlorpromazine-equivalent dose of antipsychotics. typCPZeq: Chlorpromazine-equivalent dose of typical antipsychotics. atypCPZeq: Chlorpromazine-equivalent dose of atypical antipsychotics. Significant p values are underlined.

### Comparison in personality assessed with SPQ

Cronbach's α values for the SPQ indices in patients with schizophrenia and healthy controls were 0.92 and 0.89 for the cognitive-perceptual factor, 0.91 and 0.90 for the interpersonal factor, 0.86 and 0.83 for the disorganized factor, and 0.95 and 0.93 for the total SPQ score, respectively, indicating high internal consistency for all indices.

SPQ scores for patients and controls are shown in Table 2. As expected, all SPQ indices were significantly higher for patients than controls, (p<0.001 for all comparisons). When the SPQ indices of the SA and nSA groups were compared using an ANCOVA, controlling for age and sex (Table 2), the total SPQ score and the interpersonal and disorganized factors were significantly higher in the SA group than the nSA group (nSA<SA, p<0.01 for these comparisons). The cognitive-perceptual factor was also higher in the SA group than in the nSA group, at a trend level (p = 0.072).

**Table 2:** Comparisons of the Schizotypal Personality Questionnaire (SPQ) scores between patients with schizophrenia with or without suicide attempts, and healthy controls.

	Patients with schizophrenia			Healthy controls	Analyses	
	Suicide attempt	No suicide attempt	Total		Analysis of covariance^a^	Analysis of covariance^a^
	(SA; n = 30)	(nSA; n = 57)	(n = 87)	(HC; n = 322)	(Total patients vs. controls)	(SA vs. nSA)
The Schizotypal Personality Questionnaire (SPQ)						
Total SPQ score	36.6±12.5	26.6±16.8	30.1±16.1	13.0±10.2	F(1, 405) = 140.0, p≤ 0.001, partial *η* ^2^ = 0.26	F(1, 83) = 8.3, p = 0.005, partial *η* ^2^ = 0.09
Cognitive-perceptual factor	15.0±7.7	11.3±8.5	12.6±8.4	3.8±3.9	F(1, 405) = 194.5, p≤ 0.001, partial *η* ^2^ = 0.32	F(1, 83) = 3.3, p = 0.072, partial *η* ^2^ = 0.04
Interpersonal factor	17.4±6.9	12.6±8.1	14.2±8.0	7.0±6.1	F(1, 405) = 81.6, p≤ 0.001, partial *η* ^2^ = 0.17	F(1, 83) = 8.6, p = 0.004, partial *η* ^2^ = 0.09
Disorganized factor	8.7±3.6	5.8±4.4	6.8±4.3	3.5±3.3	F(1, 405) = 56.0, p≤ 0.001, partial *η* ^2^ = 0.12	F(1, 83) = 9.4, p = 0.003, partial *η* ^2^ = 0.10

Significant p values are underlined.

a Age and sex were controlled for.

The ROC analysis showed that a total SPQ score of 33.5 was the optimal cut-off value to discriminate the SA group from the nSA group. High schizotypy (SPQ≥34) was significantly associated with the SA group (Odds ratio: 4.7, 95% confidence interval: 1.8–12.1, χ^2^[1] = 10.6, p = 0.002) (Table 3).

**Table 3:** Distributions of schizophrenia patients, with and without suicide attempts, scoring above and below the cut-off on the SPQ total score.

		SPQ total score	
		SPQ≤33	SPQ≥34
Patient groups	SA (n = 30)	9	21
	nSA (n = 57)	38	19
	Total	47	40

Odds ratio = 4.7 (95% confidence interval: 1.8–12.1), Sensitivity = 0.70, Specificity = 0.67.

SPQ, Schizotypal Personality Questionnaire; SA, patients with schizophrenia and a history of suicide attempt; nSA, patients with schizophrenia without a history of suicide attempt.

## Discussion

Significant differences in the SPQ total score and interpersonal and disorganized factors were found between the SA and nSA groups. ROC analysis showed that 33.5 was the optimal SPQ total score cut-off value to discriminate the SA group from the nSA group. These findings supported hypothesis. Patients with schizophrenia scored substantially higher on SPQ indices than controls. Nonetheless, there were still significant differences among patients on the indices based on the history of suicide attempts.

Few studies have examined schizotypal traits assessed by the SPQ in patients with schizophrenia [22], [25], [26], and to our knowledge, this is the first report showing significant differences on the total score and interpersonal and disorganized factors of the SPQ between schizophrenia patients with and without suicide attempts. Previous studies have reported that individuals with SPD who score high on the SPQ, which was developed in accordance with the criteria for SPD, are at high risk for suicide attempts and completed suicide [20], [38]. Bornstein et al. compared the clinical characteristics of SPD and non-SPD outpatients who sought psychiatric services and found that SPD was associated with a significant risk of attempted suicide [20]. In addition, Fenton et al. reported that the rates of suicide attempts and suicidal ideation in patients with SPD were 24% and 45%, respectively [38]. This evidence is compatible with the observed association between SPQ scores and suicide attempts in patients with schizophrenia, suggesting support for a common suicidality mechanism in individuals with greater SPQ scores across schizophrenia spectrum disorders.

Regarding the association between suicide attempts and the symptoms of schizophrenia, there was no difference between the SA and nSA groups on the PANSS indices. Previous studies have reported no association between suicidal behavior and positive or negative symptoms. Grunebaum et al. reported no association between the presence of delusions and the history of suicide attempts in major depressive disorder, bipolar disorder, or schizophrenia [39]. In contrast, it has been reported that command hallucinations could be a risk factor for suicide attempts in patients with a history of suicide attempts [40]. In a meta-analysis [5], hallucination was associated with decreased risk of suicide. In our study, the SA and nSA sample sizes were not large, which may have led to a type II error. Therefore, further research with larger sample sizes is warranted to elucidate the relationship between suicide attempts and the symptoms of schizophrenia. On the other hand, as the mean total PANSS score was higher in the SA group compared to the nSA group (albeit not significantly different), it may be that symptom severity (PANSS score) confounds the observed relationship between high SPQ indices and suicide attempts. To address the issue, we performed ANCOVAs to compare SPQ indices between the two groups, controlling for the total PANSS score, as well as age and sex. The obtained p values for the total SPQ score, interpersonal, and disorganized factors remained significant (F[1, 82] = 6.8, p = 0.01, F[1, 82] = 7.1, p = 0.009 and F[1, 82] = 8.0, p = 0.006, respectively).

Although the present study was not a longitudinal one, it is theoretically unlikely that suicidality leads to schizotypy. Therefore, our findings suggest that schizotypy leads to suicidality in patients with schizophrenia. Schizotypy may be associated with suicide attempts in psychiatric disorders other than schizophrenia. The comorbidity of cluster A personality disorders reportedly increases the risk for suicide attempts in patients with major depressive disorder [41] and bipolar disorder [42], but not in patients with alcohol dependence [43]. Interestingly, previous studies, including our earlier study, have reported a positive correlation between SPQ indices and harm avoidance assessed on the Temperament and Character Inventory [23], [44]; the harm avoidance temperament is associated with suicide attempts in major depressive disorder [45] and bipolar disorder [46]. Taken together, the evidence suggests that SPQ indices are useful to assess the risk of suicide attempt in patients with major depressive disorder or bipolar disorder, which warrants future research.

There are some limitations to this study. First, the cross-sectional nature of this study prevents causal interpretation. We could not determine whether the observed differences in SPQ indices between the SA and nSA groups were a cause or result of suicidal behavior. Second, given that medications such as antipsychotics have been shown to have some effect on personality traits [47], the possible effects of medication on the personality traits observed in this study cannot be ruled out, although there was no significant difference between the SA and nSA groups in the daily dose of antipsychotic medications. Third, Japan has one of the highest suicide mortality rates in the general population among developed countries [48], which is a major public health problem. Further research across cultures is needed to confirm the results. Finally, the control group would have suffered range-restriction on schizotypy. Therefore, our results need to be replicated using other study designs, such as a population-based study with subjects with a wider range of scores on the SPQ indices.

In summary, significant differences were found for the total score and interpersonal and disorganized factors of the SPQ between schizophrenia patients with and without a history of suicide attempts, and on all SPQ indices between patients with schizophrenia and controls. The cut-off value of 33.5 for the total SPQ score discriminated the SA group from the nSA group, with a high odds ratio of 4.7. This suggests that the SPQ is a useful supplement to existing screening methods for suicide risk in schizophrenia.
